# Mechanisms of Oral Bacterial Virulence Factors in Pancreatic Cancer

**DOI:** 10.3389/fcimb.2019.00412

**Published:** 2019-12-04

**Authors:** Zhong Sun, ChengLong Xiong, Seoh Wei Teh, Jonathan Chee Woei Lim, Suresh Kumar, Karuppiah Thilakavathy

**Affiliations:** ^1^Department of Biomedical Science, Universiti Putra Malaysia, Serdang, Malaysia; ^2^Department of Public Health Microbiology, School of Public Health, Fudan University, Shanghai, China; ^3^Department of Medical Microbiology and Parasitology, Universiti Putra Malaysia, Serdang, Malaysia; ^4^Pharmacotherapeutics Unit, Department of Medicine, Faculty of Medicine and Health Sciences, Universiti Putra Malaysia, Serdang, Malaysia; ^5^Genetics and Regenerative Medicine Research Centre, Universiti Putra Malaysia, Serdang, Malaysia; ^6^UPM-MAKNA Cancer Research Laboratory, Institute of Bioscience, Universiti Putra Malaysia, Serdang, Malaysia

**Keywords:** oral bacteria, pancreatic cancer, virulence factors, CDT, FadA, NDK, Gingipains

## Abstract

Pancreatic cancer is a highly lethal disease, and most patients remain asymptomatic until the disease enters advanced stages. There is lack of knowledge in the pathogenesis, effective prevention and early diagnosis of pancreatic cancer. Recently, bacteria were found in pancreatic tissue that has been considered sterile before. The distribution of flora in pancreatic cancer tissue was reported to be different from normal pancreatic tissue. These abnormally distributed bacteria may be the risk factors for inducing pancreatic cancer. Therefore, studies on combined effect of multi-bacterial and multi-virulence factors may add to the knowledge of pancreatic cancer pathogenesis and aid in designing new preventive and therapeutic strategies. In this review, we outlined three oral bacteria associated with pancreatic cancer and their virulence factors linked with cancer.

## Introduction

Pancreatic cancer is considered as one of the deadliest cancers, since its onset is occult and the early symptoms are not typical (Kamisawa et al., 2016). Although the detection and treatment of pancreatic cancer has progressed, the 5-year survival rate of pancreatic cancer is only 9%, which is the lowest among all cancers (Siegel et al., 2019). Worldwide, the incidence of pancreatic cancer is increasing year by year. There are 458,918 new cases in 2018, which means more than 1,250 people are told to have pancreatic cancer every day. In the same year, 432,242 patients died of pancreatic cancer, therefore, it has become the seventh leading cause of cancer-related deaths (Rawla et al., 2019). The reason for this situation is that researchers do not know enough about the pathogenesis of pancreatic cancer.

According to the 2018 International Agency for Research on Cancer (IARC) ~18% of cancers are associated with infectious diseases caused by bacteria, viruses and parasites (Rawla et al., 2019). Bacterial infections promote the formation of inflammatory microenvironment, which is a critical regulator of carcinogenesis (Coussens and Werb, 2002). Persistent infections will induce epigenetic modification of the somatic cells and lead to the production of a large amount of reactive oxygen species (ROS) and reactive nitrogen (RNS), that eventually cause DNA damage, oncogene activation or tumor suppressor genes inactivation (Cuevas-Ramos et al., 2010; Sahan et al., 2018).

## Bacteria and Pancreatic Cancer

The pancreas has been considered as sterile for a long time due to presence of highly alkaline pancreatic enzymes. However, it was found that microorganisms can reach pancreas through blood and digestive systems (Michaud, 2013). Recent studies have reported that the content and composition of bacteria and fungi in pancreatic cancer tissues are different from normal pancreatic tissues. Their presence not only promote the occurrence of pancreatic cancer, but also affects its prognosis (Pushalkar et al., 2018; Aykut et al., 2019; Riquelme et al., 2019). Pushalkar et al. reported that *Bacteroidetes* (31%) and *Firmicutes* (22%) dominated the healthy pancreas, whereas *Proteobacteria* found to be abundant in pancreatic cancer tissues and was associated with advanced disease. *Actinobacteria* (1%) was also reported present in pancreatic cancer tissue although the abundance was low (Pushalkar et al., 2018). On the same year, Fan et al. conducted prospective study on the relationship between oral microbes and pancreatic cancer, and found that *Aggregatibacter actinomycetemcomitans* (*A. actinomycetemcomitans*) and *Porphyromonas gingivalis* (*P. gingivalis*), and decreased relative abundance of *Fusobacterium nucleatum* (*F. nucleatum*) are associated with onset risk of pancreatic cancer (Fan et al., 2018). However, in another study, *F. nucleatum* was independently associated with a poor prognosis for pancreatic cancer (Mitsuhashi et al., 2015). The results of these experiments showed that the abundance variation of these three bacteria were associated with increased risk of pancreatic cancer. In recent years, prospective cohort studies and case-control studies could not conclude that *Helicobacter pylori* (*H. pylori*) is associated with increased risk of pancreatic cancer (Wei et al., 2019). Moreover, *H. pylori* cannot be detected in chronic pancreatitis and pancreatic cancer tissues, which proved that it could not directly participate in the development of cancer (Jesnowski et al., 2010). However, the effect of *H. pylori* on gastric mucosa and suppression of gastric acid secretion, which resulting low acidity in the stomach may provide other oral bacteria an opportunity to enter the pancreas (Michaud, 2013). Since there is association among bacteria in cancer development, Tjaslsma et al. proposed a “driver-passenger” model according to characteristics of the participating microbes (Tjalsma et al., 2012). Based on these studies, we have selected several digestive tract bacteria that may be involved in the pathogenesis of pancreatic cancer (Table 1).

**Table 1:** The characteristics of cancer causing digestive tract bacteria and their correlation with pancreatic cancer.

	***H. pylori***	***A. actinomycetemcomitans***	***F. nucleatum***	***P. gingivalis***
Phylum	*Proteobacteria*	*Proteobacteria*	*Fusobacteria*	*Bacteroidetes*
Gram stain	Negative	Negative	Negative	Negative
Respiration characteristic	Microaerophilic	Facultative anaerobe	Anaerobic	Anaerobic
Location	Stomach	Oral	Oral	Oral
Pancreatic tissue	Absence	Presence	Presence	Presence
Correlation with pancreatic cancer	Poor	High	High	High

## Oral Bacterial Virulence Factors and Cancer

Virulence factors produced by oral bacteria assist them to invade the host and cause diseases. Primarily, they are causal agents of periodontitis (Shang et al., 2019). However, studies have also identified pathogenic components of oral bacteria as significant risk factors for developing other diseases. For example, *A. actinomycetemcomitans* is associated with endocarditis, rheumatoid arthritis (Paturel et al., 2004; Mukherjee et al., 2018), *F. nucleatum* is associated with colorectal carcinoma (Repass et al., 2018), and *P. gingivalis* is associated with the onset of Alzheimer disease, atherosclerosis and diabetes (Sugiyama et al., 2012; Velsko et al., 2014; Laugisch et al., 2018). This paper reviews the virulence factors of the three oral bacteria and their mechanisms of action associated with cancer, particularly pancreatic cancer.

## Aggregatibacter actinomycetemcomitans

*Aggregatibacter actinomycetemcomitans* is a Gram-negative, facultative anaerobe, non-motile bacterium which can enter the host cells by endocytosis, and then secrete phospholipase C to destroy membrane vesicles and release themselves into the cytoplasm. However, the invasion is a dynamic process, after growth and division, which anchors to the host cell membrane and enters adjacent epithelial cells or deep cells through microtubules. This process benefits *A. actinomycetemcomitans* to infect deep cells and escape from immune system (Henderson et al., 2010).

*Aggregatibacter actinomycetemcomitans* can secrete a variety of toxins, of which the following three are most studied. The first variety is Leukotoxin (LtxA), a lipoprotein belonging to the RTX family of toxins, attached to neutroplils, monocytes and lymphocytes. It will form pores on their cell membrane, thereby altering its function of osmotic homeostasis, leading to cell death (Johansson, 2011).

The second type is cell cytolethal distending toxins (CDT), a bacterial toxin of AB2 trimer, which is made of the active subunit (CdtB) and two binding subunits (CdtA and CdtC). The enzymatically active subunit CdtB has structural and functional homology similar with mammalian deoxyribonuclease I (DNase I). CDTis the only member of AB toxin family with DNase enzyme activity. The role of CdtA and CdtC is to anchor CdtB on host cell membrane, in which CdtC is considered to be a chaperone for CdtB. After entering cell by endocytosis, CdtB undergoes retrograde translocate to endoplasmic reticulum (ER) via Golgi complex, then directly trans from ER to nucleus (Frisan, 2015). Due to its potent DNase activity, CDT is sufficient to induce DNA damage at very low doses (50 pg / mL). Single-strand breaks (SSB) can be induced after CDT intervention in 3 h. SSB causes replication forks (RFs) to stall, producing replication stress response, that ultimately leads to double-strand breaks (DSB) and cell cycle arrest. However, high doses (4 μg/ mL) of CDT can directly lead to important levels of DSB (Jinadasa et al., 2011).

DSB in host cells can be recognized by MRN complex (composed of MRE11, RAD50, and NBS1). The latter recruits ATM (ataxia-telangiectasia mutated) kinase to DNA injury sites, then ATM phosphorylates the CHK2 transduction proteins (Lee and Paull, 2005). Activated ATM and CHK2 phosphorylate a variety of substrates, including the p53 and CDC25 phosphatase families, while effector protein (p53, CDC25) activates appropriate cellular response (Jazayeri et al., 2006). These cellular responses include the following: (1) ATM-dependent DNA damage response (DDR): With the advent of DBS, DNA repair mechanisms are initiated, DDR is activated, including homologous recombination (HR) and non-homologous end joining (NHEJ) repair mechanism (Goodarzi and Jeggo, 2013). (2) ATM-dependent cell cycle arrest: Activated ATM phosphorylates p53, resulting p21 upregulates cytosine E-CDK2, which blocks cells from entering the S phase (Mediates G1/S blockade). Activated Chk2 phosphorylates and inactivates cell division cycle 25 (CDC25) C phosphatase, resulting accumulation of phosphorylated cyclin B-CDK1 complex, which prevents cells from entering the M phase (Mediated G2/M blockade) (Jinadasa et al., 2011). These can lead to cell cycle arrest, resulting in the formation of corresponding tissue microenvironment, that not only promotes survival and proliferation of transformed cells through by senescence-associated secretory phenotype (SASP) but also promote cancer occurrence (Coppé et al., 2010; Campisi, 2013).

In some cases, genetic instability caused by improper DNA damage repair is a significant reason of cancer development. In the other cases, CDT can also induce apoptosis. When DDR systems fail to properly repair DNA damage, thereby activating p53, leading to activation of intrinsic apoptotic pathway, which ultimately leads apoptotic cell to death (Jinadasa et al., 2011). At the same time, CdtB has phosphatase activity, which can decompose PI-3,4,5-P_3_ (PIP3) to PI-3,4-P_2_, thereby changing pathway of PI-3K/PIP_3_/AKT/pGSK3β signaling, leading inactivation of the downstream Akt pathway, which ultimately leads to cell cycle arrest and activation of the apoptosis cascade (Shenker et al., 2007). The PIP3 is mainly synthesized by PI-3Ks intracellular. Accompanied by a large consumption of PIP3, which leads to excessive activation of PI-3K. Since PI-3K is one of the major effectors of KRAS, activation of KRAS signaling by RAC1 via PI-3K is required for KRAS mutation. It is well-known that almost all pancreatic ductal adenocarcinoma (PDA) has mutations in the KRAS gene (Wu et al., 2014). Therefore, CDT can also modulate cyclomodulin in addition to genotoxin.

**Figure 1:**
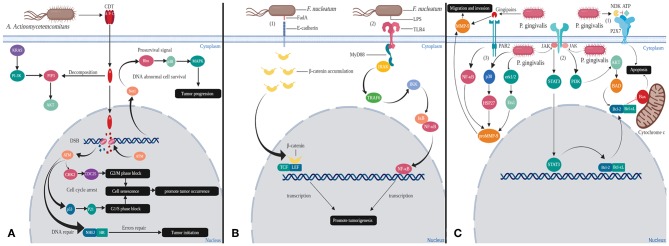
Mechanisms of oral bacteria virulence factors inducing changes in host cells. **(A)** Cytolethal distending toxins (CDT) are the virulence factors released by *A. actinomycetemcomitans*: In the cytoplasm, the phosphatase activity of CdtB can decompose PIP3, thereby over-activating PI-3K, which is one of effectors of KRAS. This process may cause KRAS mutation that leads to cancer. In the nucleus, CdtB causes double strand break (DSB), which activates ataxia telangiectasia mutated (ATM) kinase. Activation of ATM kinase blocks G1/S and G2/M phases promoting tumor occurrence through cell senescence. Tumor initiation also could occur in the instance of erroneous in homologous recombination (HR) and non-homologous end joining (NHEJ) repair mechanisms. In order for the cells to survive, RhoA and p38 MAPK will get activated, thereby promoting tumorigenesis. **(B)** FadA and LPS are the significant virulence factors of *F. nucleatum*. (1) Binding of FadA to host cell E-cadherin causes accumulation of β-catenin in cytoplasm that eventually enters into nucleus. β-catenin will act together with LEF/TCF and produce abnormal proteins, which ultimately leads to cancer. (2) LPS binds to host cell TLR4 receptor and induces MyD88 recruitment. These will activate NF-κB signaling pathway to direct cell proliferation and cancer development. **(C)** Gingipains and NDK are the virulence factors secreted by *P. gingivalis*. (1) NDK can decompose ATP and inhibit p2x7-mediated apoptosis. (2) Gingipains able to upregulate matrix metalloproteinase 9 (MMP-9) outside the cells and proMMP-9 via NF-kB pathways in the cytoplasm that contribute to the metastasis of cancer cells. (3) *P. gingivalis* also could enter the cells and increase the expression of proMMP-9 by activating erk1/2-ets1, p38/HSP27. Moreover, *P. gingivalis* invasion could inhibit release of cytochrome c and activate caspase-9 and caspase-3 by dual JAK/Stat and Akt signaling, thereby allowing damaged or diseased cells to survive.

The fate of cells after CDT infection seems to depend on the cell type. When lymphocytes are present due to antigen presentation or mitosis, the PIP3 content is increased. The phosphatase activity of CdtB is enhanced with the increase of intracellular PIP3 concentration. Lymphocytes undergo cell cycle arrest or apoptosis, which ultimately impair host immunity and conducive to the formation of chronic infection microenvironment (Shenker et al., 2007). In fact, almost all the host cells will undergo cell cycle arrest after infection with CDT. Among them, hematopoietic cell lineage will progress toward apoptosis, while epithelial and mesenchymal cell lineages will remain alive, which is associated with activation of the survival signal transduction pathway in these adherent cells. In order to resist the toxin action of CDT, Net1 (transforming gene 1) regulates the activation of ras homolog family member A (RhoA) and p38 mitogen-activated protein kinase (MAPK). Activation of the survival signal causes these cells to survive, and also allows cells with incorrect DNA repair to survive, thereby promoting tumorigenesis and progression (Guerra et al., 2008) (Figure 1A).

Based on the above CDT mechanism of actions some researches have confirmed that CDT can promote the occurrence of liver cancer and colorectal cancer (Ge et al., 2007; Graillot et al., 2016).

The third type of cytotoxin is cytotoxin-associated gene E (CagE). CagE may have helicase activity due to the presence of DEAD cassette, and its role in regulating DNA methylation expression is considered as possible mechanisms of tumorigenesis. Because of above functions, CagE can participate in a variety of cellular activities, including mutagenesis, senescence and regulation of tissue-specific gene expression. Therefore, the CagE gene can be widely expressed in various cancer cell lines and cancer tissues including pancreatic cancer (Kim and Jeoung, 2008). In fact, it shares homology with the carcinogenic factors secreted by *H. pylori* (Henderson et al., 2010).

## Fusobacterium nucleatum

*F. nucleatum* is a Gram-negative anaerobic bacterium, but unlike many strict anaerobic bacteria, possess NADH oxidase, endowing them with a limited ability to respire oxygen (Kapatral et al., 2002). *F. nucleatum* is an adherent bacterium that encodes a variety of adhesins so that it can copolymerize various microorganisms. At the same time, its main virulence factors are also adhesion proteins from the outer membrane. Among all the adhesins, only *Fusobacterium* adhesin A (FadA), was identified as capable of binding to host cells and is also the most characteristic virulence factor of *F. nucleatum*. FadA exists in two forms, non-secretory intact pre-FadA and secreted mature FadA (mFadA). When pre-FadA mixed with mFadA, they form activity complexes, FadAc, which binds to host's receptor for attachment and invasion. The host receptors for FadA are members of the cadherin family, mainly E-cadherin and vascular endothelial (VE) cadherin (CDH5), which are required for *F. nucleatum* adhesion and invasion (Fardini et al., 2011; Rubinstein et al., 2013). FadA binds to E-cadherin of epithelial cells, resulting in phosphorylation and internalization of E-cadherin on the membrane. Subsequently, canonical Wnt pathway is activated, accompanied by decreased phosphorylation of β-catenin, which accumulates in the cytoplasm and translocates to the nucleus (Rubinstein et al., 2013). This process results in activation of β-catenin-regulated transcription (CRT), which interacts with transcription factors lymphoid enhancer factor (LEF)/T-cell factor (TCF). Eventually the Wnt target gene *c-myc* and *cyclin D1* are transcribed (Figure 1B). In general, Wnt signaling pathways can regulate cell differentiation and proliferation, thus also involve many aspects of pancreatic biology. Studies have shown that during the development of pancreatic cancer, Wnt signaling activity is gradually increased, and activation of the Wnt/β-catenin pathway is necessary for the initiation of pancreatic cancer (Rubinstein et al., 2013; Zhang et al., 2013). In addition, FadA binds to VE-cadherin on vascular endothelial cells, causing the latter to migrate from cell-cell junction to intracellular compartment, increasing endothelial permeability. Therefore, FadA not only directly invades host cells but also allow invasion of itself and other bacteria into blood by increasing endothelial permeability, which is conducive to spread of infection and immune escape (Fardini et al., 2011; Rubinstein et al., 2013).

Another virulence factor of *F. nucleatum* is familial adenomatous polyposis 2 (Fap2), which is an outer membrane protein. Fap2 binds and interacts to human inhibitory receptor T cell immunoreceptor with Ig and ITIM domains (TIGIT), that is present on human natural killer (NK) cells and lymphocytes. The cytotoxic effects of NK cells and lymphocytes are suppressed, which ultimately protecting tumor from immune system and promoting the formation of inflammatory microenvironment (Gur et al., 2015).

Moreover, during the infection invasion of *F. nucleatum*, after binding with host cell toll-like receptor 4 (TLR4) receptor, lipopoly-saccharide (LPS) of *F. nucleatum* interacts with Toll/ il-1 receptor (TIR) domain-containing adaptor inducing IFN-beta (TRIF) to induce myeloid differentiation primary response protein 88 (MyD88) recruitment. MyD88 induces IRAK (IL-1 receptor–associated kinase) phosphorylation, which dissociates from the receptor, interacts with adaptor proteins TNFR-associated factor 6 (TRAF6) and TAK1 -binding proteins 2 (TAB2) on the membrane, and regulates their transport to the cytosol. Subsequently, TRAF6 becomes ubiquitinated (Ub) and activates TAK1 (TGF-β-activated kinase 1), the latter phosphorylates and activates the IκB kinase (IKK) complex. IKK phosphorylates IκB, an inhibitor of nuclear factor kappa B (NF-κB), which allows NF-κB to be rapidly activated and transferred to nucleus, promoting expression of related genes by binding to κB (Janssens and Beyaert, 2002; Wu et al., 2018). NF-κB is a multifunctional dimeric transcription factor that coordinates cell proliferation and closely related to cancer development and progression (Yang et al., 2017) (Figure 1B). In addition, it has been reported that high levels of MyD88 promote PDAC cell growth and are associated with low survival in patients with PDAC (Yang et al., 2017).

## Porphyromonas gingivalis

*P. gingivalis*, a Gram negative anaerobeis, can replicate to high levels after invading host cells without inducing host cell death, thereby contributing to extend their common survival time (Yang et al., 2017). The major virulence mechanism of *P. gingivalis* involve three pathways as follows:

In the first pathway, the nucleoside diphosphate kinase (NDK) secreted by *P. gingivalis* can act as an ATPase to reduce ATP concentration and inhibit apoptosis (Yilmaz et al., 2008) (Figure 1C). The purinergic receptor P2X7, located in cell membrane, mediates ATP-dependent apoptosis, which is considered to be a cytotoxic receptor. Actually, low levels of ATP promote cell growth and proliferation, high doses of ATP cause cell death (Adinolfi et al., 2005).

In the second pathway, *P. gingivalis* activates various anti-apoptotic/pro-survival pathways and keeps host cells survival by partially blocking mitochondrion-dependent apoptosis. These activation pathways include both of JAK/PI3K/Akt and JAK/STAT3. When cells are stimulated by canceration, dephosphorylated, Bcl-2-antagonist of cell death (BAD) forms a heterodimer with B-cell lymphoma 2 (Bcl-2) and B-cell lymphoma-extra large (Bcl-xL), thereby inactivate the latter, allowing Bax/Bak to form pores in the outer membrane of mitochondria. The above process causes cytochrome c (Cyt c) to leak from the mitochondria into cytoplasm and activate the pro-apoptotic caspase cascade to initiate apoptosis. *P. gingivalis* can activate the JAK/PI3K/Akt signaling pathway, in which Akt can phosphorylate BAD and forms a BAD-(14-3-3) protein heterodimer. This process allows Bcl-2 to freely inhibit Bax-triggered apoptosis (Yilmaz et al., 2004; Mao et al., 2007). In addition, *P. gingivalis* can simultaneously activate JAK/STAT3 signaling pathway and up-regulate miR-203, which inhibits the negative regulatory factor suppressor of cytokine signaling 3 (SOCS3). When SOCS3 is inhibited, STAT3 activity is significantly enhanced, that targets mainly anti-apoptotic genes (such as Bcl-2, Bcl-XL) (Yasukawa et al., 2003; Moffatt and Lamont, 2011; Bousoik and Aliabadi, 2018). Thus, the combination of these pathways results in up-regulation of anti-apoptotic Bcl-2 and down-regulation of pro-apoptotic Bax. Anti-apoptotic Bcl-2 and Bcl-xL proteins not only inhibit Cyt c release through mitochondrial pores, but also inhibit Cyt c activation of the cytoplasmic caspase cascade, ultimately promoting host cell survival and proliferation (Mao et al., 2007) (Figure 1C). Ikezawa *et al*. showed that overexpression of Bcl-xL is present in 90% of pancreatic ductal adenocarcinoma (PDAC) (Ikezawa et al., 2017).

Additionally, gingipains secreted by *P. gingivalis* stimulate proteinase-activated receptor 2 (PAR2) and then activate the pathway of PAR2/NF-κB. Meanwhile, *P. gingivalis* can activate erk1/2 -Ets1 and p38/HSP27 pathways after invading host cells. The above three pathways jointly induce the expression of promatrix metalloproteinase (proMMP-9). Thereafter, proMMP9 is released into extracellular environment by host cells via PAR2 activated by gingipains. Activated MMP-9 can degrade a variety of extracellular matrices (ECM) by proteolytic cleavage. Destruction of ECM is often a necessary step during tumor invasion and metastasis (Inaba et al., 2014; Whitmore and Lamont, 2014). Studies have shown that MMP-9 is overexpressed in PDAC and plays an important role in the invasion and metastasis (Ikezawa et al., 2017) (Figure 1C).

## Conclusion

In the process of inducing cancer, bacteria act as a team. This mode of cooperation has been summarized as bacterial driver-passenger model, where the initial pathogen known as “driver,” e.g., *A. actinomycetemcomitans*, induce DNA damage in host cells. This driver pathogen causes microenvironment changes around the host cell, which facilitates proliferation and survival of other pathogens resulting in a more stable ecosystem. Then the “passenger” bacteria, e.g., *F. nucleatum* will locate the cancer cells and act as bridging organism between the early (*A. actinomycetemcomitans*) and late colonizing microbes (*P. gingivalis*). *P. gingivalis* can inhibit cancer cell apoptosis and promote tumor development. The three bacteria may play an important synergistic role in the occurrence and development of cancer. Therefore, the diverse microbial ecosystem is not only more stable than single type of bacteria but also more toxic, which might be one of the important factors in inducing cancer. In conclusion, with deepening of research on relationship between bacteria and cancer, uncovering the mechanisms of bacterial cooperation may bring new dawn to early diagnosis and treatment of pancreatic cancer.

## Author Contributions

All authors have made a significant, direct and intellectual contribution to this manuscript. ZS designed, drafted and edited the manuscript. CX revised the figures and the manuscript. JL revised and edited the manuscript. ST edited the manuscript. SK conceptualized the study, reviewed and edited the manuscript. KT conceptualized and designed the study and reviewed and edited the manuscript. All authors approved the final manuscript as well as the authorship list.

### Conflict of Interest

The authors declare that the research was conducted in the absence of any commercial or financial relationships that could be construed as a potential conflict of interest.
